# Differences of Hemogram Parameters and Their Ratios among Patients with Takotsubo Syndrome, Acute Coronary Syndrome and Healthy Individuals

**DOI:** 10.3390/life12060788

**Published:** 2022-05-26

**Authors:** Albert Topf, Moritz Mirna, Nina Bacher, Lukas Schmutzler, Peter Jirak, Bernhard Ohnewein, Uta C. Hoppe, Michael Lichtenauer

**Affiliations:** Clinic for Internal Medicine II, University Hospital Salzburg, Paracelsus Medical University, Müllner Hauptstraße 48, A-5020 Salzburg, Austria; m.mirna@salk.at (M.M.); n.bacher@salk.at (N.B.); l.schmutzler5@gmail.com (L.S.); p.jirak@salk.at (P.J.); bernhardoh@icloud.com (B.O.); u.hoppe@salk.at (U.C.H.); m.lichtenauer@salk.at (M.L.)

**Keywords:** Takotsubo syndrome, hemogram parameters, acute coronary syndrome

## Abstract

Introduction: Takotsubo cardiomyopathy (TTC) and acute coronary syndrome (ACS) are clinically indistinguishable from each other. Although therapeutically redundant, coronary angiography remains indispensable for differential diagnosis. Methods: In our study, we compared hemogram parameters and their ratios in 103 patients presenting with undiagnosed chest pain. Blood was drawn at baseline in 40 patients with TTC, 63 patients with ACS, and 68 healthy controls ((Ctrl) no coronary artery disease or signs of heart failure). Results: Peripheral lymphocyte counts were significantly depressed in TTC and ACS patients when compared to the Ctrl. Consequently, all three investigated hemogram ratios were significantly elevated in patients with ACS or TTC (NLR: TTC: median 3.20 vs. ACS: median 3.82 vs. Ctrl: median 2.10, *p* < 0.0001; BLR: median 0.02 vs. ACS: median 0.00 vs. Ctrl: median 0.00, *p* < 0.0001; MLR: median 0.37 vs. ACS: median 0.44 vs. Ctrl: median 0.28, *p* < 0.0001). Of note, BLR was only significantly elevated in patients with TTC, and not in patients with ACS (ACS vs. Ctrl *p* = 0.183). Conclusion: Basophil count and BLR are significantly increased in TTC patients when compared to ACS and may, therefore, be helpful in the distinction of TTC from ACS. Whereas NLR might be useful to differentiate ACS from controls. Elevated basophil counts and BLR in TTC patients are interesting findings and may confirm speculations about the partly unexplained pathophysiology.

## 1. Introduction

Takotsubo cardiomyopathy constitutes a form of cardiomyopathy with transient left ventricular dysfunction. Clinical symptoms, electrocardiographic alterations, and changes in cardiac biomarkers may be similar to acute coronary syndrome (ACS) [1]. Approximately 1–2% of all patients and up to 10% of women with suspected acute coronary syndrome (ACS) are later diagnosed with TTC [2]. TTC predominantly affects postmenopausal women and is usually triggered by physical or emotional stress factors. The left ventricle typically demonstrates apical ballooning and basal hypercontractility but also midventricular, basal, as well as focal left ventricular contractile abnormalities can be seen [3]. Inflammation, sympathetic overdrive-mediated multi-vessel epicardial spasms, microvascular dysfunction, and direct toxicity of catecholamines have been proposed as pathophysiological mechanisms [4]. The pathophysiology of ACS may be heterogeneous too, but in the majority, it is associated with rupture of an atherosclerotic plaque and partial or complete thrombosis of the culprit lesion. Ruptured plaques contribute to thrombus formation by inflammatory mechanisms, leading to the development of ACS [5].

At present, a distinction between ACS and TTC based solely on clinical presentation and routine cardiovascular biomarkers is not possible. Scores and novel biomarkers have been investigated for their differential diagnostic value in clinical studies, however, so far, there has been no clinical implementation. Coronary angiography still remains necessary for differential diagnosis [6]. Hemogram parameters and ratios have already been analyzed for their prognostic value in ACS and TTC [7,8]. However, there is no publication investigating the differential diagnostic value of hemogram parameters and their ratios.

Leukocyte count has been identified to be involved in the pathogenesis of coronary heart disease in the 1920s [9]. Similarly, lymphocytes, as the regulatory arm of the immune system, have been investigated to be important in inflammatory processes at various stages of the atherosclerotic process. Lymphopenia is indicative of worse clinical outcomes after ST-elevation ACS [10]. Neutrophils have been found to be the first leukocytes in damaged myocardial structure. Neutrophils have been reported to be predictive of long-term outcomes after ACS. Similar to neutrophil counts, the neutrophil-to-lymphocyte ratio (NLR) shows prognostic relevance in many diseases, such as malignancies, infectious pathologies, ACS, and myocarditis [11]. Furthermore, NLR presented as an independent predictor of in-hospital complications in TTC patients [12]. Eosinophils are suspected to be associated with protective inflammatory responses following myocardial tissue injury and that increased eosinophil counts are predictive of all cause-death after elective or urgent PCI [13]. Basophils have a life span of 2–3 days and are rapidly mobilized by the inflammatory state. Basophils produce high-affinity IgE and are known to secrete proteolytic enzymes, and bioactive lipids (PGE, Leukotriene, histamine, TNF alpha, IL-6, IL-4and IL-13). Basophils are leucocytes with proinflammatory effects. Low basophil levels seem to be associated with worse outcomes in patients with ACS [14]. ELR and BLR are other rates that present the inflammatory state. Eosinophils and basophils are closely related to allergic conditions. Higher ELR was found as a new inflammatory marker in smokers compared to non-smokers [15]. It has been demonstrated that NLR and ELR can be used as a marker of occult inflammation and disease activity in patients diagnosed with achalasia. In systemic autoimmune rheumatic diseases, ELR and BLR may vary depending on the disease. It has been shown that in many systemic autoimmune rheumatic diseases, BLR is decreased, while ELR is decreased in systemic lupus erythematosus and increased in other systemic autoimmune rheumatic diseases [16]. Monocytes seem to be actively involved in all phases of ACS. Monocytes should not only be considered as a classical element of innate immunity but moreover as an important part of the coagulation system and potentially as a regenerative instrument of the human species. Elevated counts of monocytes and a higher monocyte-to-lymphocyte ratio (MLR) are reported to be associated with the severity of coronary lesions. In myocarditis, MLR showed to be prognostic for the length of hospital stay [17].

After several studies investigating the prognostic impact of hemogram parameters and their ratios, we aimed to analyze those parameters for the differentiation between TTC, ACS, and healthy patients. By analyzing hemogram parameters, more information about the partly unknown pathogenesis of TTC may be revealed.

## 2. Materials and Methods

**Patients and controls.** The study was approved by the local ethics committee of Salzburg (415-E/2230/10-2018) and was performed in accordance with the Declaration of Helsinki and Good Clinical Practice. All patients provided written informed consent prior to enrollment.

In this study, we recruited 103 consecutive patients hospitalized for undiagnosed chest pain. A total of 40 patients with TTC were included if they fulfilled the Mayo Clinic Diagnostic Criteria for TTC [18]. In total, 63 patients with an ACS were enrolled. In accordance with the European Society of Cardiology criteria, ACS was diagnosed and treated [19]. We also recruited 68 healthy subjects without coronary artery disease or echocardiographic signs of heart failure for our control group.

Blood samples were collected within 24 h after the onset of symptoms and were compared within the groups. Data on clinical presentation, precipitating factors, cardiovascular risk factors, medications, and demographics were obtained as well.

**Transthoracic echocardiography.** Transthoracic echocardiography at baseline (Philips iE 33 ultrasound system, LEADS in Missouri City, TX, USA) was used to assess left ventricular ejection fraction (LVEF). Standard echocardiographic views, including the parasternal long axis view, parasternal short axis view, and apical four chamber view, were acquired as previously published [20].

**Statistical analysis.** Statistical analysis was performed using SPSS (22.0, SPSS Inc., Chicago, IL, USA). The Kolmogorov–Smirnov test was used to assess the distribution of data in the study population. As most parameters were not normally distributed, all values were given as a median and interquartile range (IQR). Median values between groups were compared by the Mann–Whitney U test or Kruskal–Wallis test with Dunn’s post hoc test. Correlation analysis was performed using Spearman’s rank correlation coefficient. ROC analysis was performed and an optimal cut-off was calculated by means of the Youden Index. Areas under the curve (AUC) were compared as described by Hanley and McNeil [21]. A *p* < 0.05 was considered to be statistically significant.

## 3. Results

**Baseline characteristics.** In total, 171 patients were enrolled in this study. Of these, 36.8% (*n* = 63) had ACS, 23.4% (*n* = 40) TTC and 39.8% (*n* = 68) constituted the control group. Baseline characteristics of enrolled patients are depicted in Table 1. In brief, patients with TTC were significantly older (median 72 years vs. ACS: 64 years vs. Ctrl: 65 years, *p* = 0.002) and had a lower BMI than patients in the ACS or control group (median 26.7 kg/m^2^ vs. ACS: 27.8 kg/m^2^ vs. Ctrl: 27.7 kg/m^2^, *p* = 0.034). Furthermore, the prevalence of female sex, diabetes mellitus, and smoking was significantly different between the three groups (see Table 1).

The left ventricular ejection fraction of patients with TTC was significantly lower than of patients with ACS or healthy controls (median 40% vs. ACS: 50% vs. Ctrl: 67%, *p* < 0.0001).

Leukocyte levels did not significantly differ among patients with TTC, ACS, and Ctrl. CRP levels were the highest in patients with ACS, with a significant difference in patients with TTC (*p* = 0.030) and a significant difference in Ctrl patients (*p* = 0.019). Regarding comorbidities, hypertension, diabetes, and smoking were more often present in Ctrl patients compared to TTC and ACS patients.

**White blood cell differentials and ratios.** Compared to healthy controls, patients with ACS and TTC showed elevated peripheral leukocyte counts (TTC: median 9.31 G/L vs. ACS: median 8.90 G/L vs. Ctrl: 6.90 G/L, *p* < 0.0001), neutrophil counts (TTC: median 5.23 G/L vs. ACS: median 6.40 G/L vs. Ctrl: 4.00 G/L, *p* = 0.002) and monocyte counts (TTC: median 0.61 G/L vs. ACS: median 0.80 G/L vs. Ctrl: 0.50 G/L, *p* < 0.0001). In contrast, peripheral lymphocyte counts were significantly depressed in these patients (TTC: median 1.50 G/L vs. ACS: median 1.60 G/L vs. Ctrl: median 1.92 G/L, *p* = 0.015).

**Figure 1:**
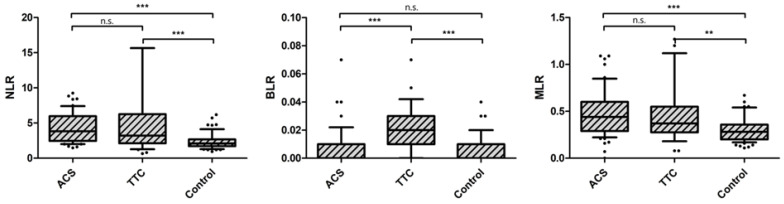
Investigated ratios between the three groups. ** indicates a *p* of <0.01 and *** a *p* of <0.001, n.s. = not significant. Abbreviations: NLR = Neutrophil/Lymphocyte ratio, BLR = Basophil/Lymphocyte ratio, MLR = Monocyte/Lymphocyte ratio.

As a consequence, all three investigated ratios were significantly elevated in patients with ACS or TTC when compared to healthy controls (NLR: TTC: median 3.20 vs. ACS: median 3.82 vs. Ctrl: median 2.10, *p* < 0.0001; BLR: median 0.02 vs. ACS: median 0.00 vs. Ctrl: median 0.00, *p* < 0.0001; MLR: median 0.37 vs. ACS: median 0.44 vs. Ctrl: median 0.28, *p* < 0.0001, see Table 2 and Figure 1).

Of note, BLR was only significantly elevated in patients with TTC, and not in patients with ACS, when compared to controls (ACS vs. Ctrl. *p* = 0.183).

**Correlation analysis in the total cohort.** Of the three investigated white blood cell ratios, MLR correlated most frequently with baseline characteristics. As such, MLR showed a significant inverse correlation with LV ejection fraction (rs = −0.208, *p* = 0.021) and significant positive correlations with age (rs = 0.192, *p* = 0.030), CRP (rs = 0.175, *p* = 0.024) and serum creatinine (rs = 0.223, *p* = 0.005). Similar to MLR, NLR also showed a significant inverse correlation with ejection fraction (rs = −0.332, *p* = 0.001). Notably, none of the three ratios correlated with BMI (see Table 3).

**Univariate logistic regression analysis.** In a reduced cohort of ACS and controls, NLR showed a significant association with the presence of ACS in univariate logistic regression analysis (B(SE) = 0.728 (0.166), Cox & Snell R^2^ = 0.24, *p* < 0.0001). This association even remained statistically significant in a multivariate model, where we corrected for possible confounders (serum creatinine, CRP, EF, age, smoking; B(SE) = 0.728 (0.166), Cox & Snell R^2^ = 0.24 *p* < 0.0001, see Supplementary Materials Table S1). Of note, since the BMI did not differ between patients with ACS and controls (BMI: *p* = 0.137) and did not show a significant correlation with the investigated ratios, we chose to not include this parameter in the multivariate model to prevent overfitting.

We then further calculated another univariate regression model in a reduced cohort of TTC and controls. Here, NLR was also predictive for the presence of TTC in univariate logistic regression analysis (B(SE) = 0.408 (0.143), Cox & Snell R^2^ = 0.18, *p* = 0.004). Again, this association even remained statistically significant in a multivariate model, where we corrected for possible confounders (serum creatinine, CRP, EF, age; B(SE) = 0.728 (0.166), Cox & Snell R^2^ = 0.24 *p* < 0.0001; see Supplementary Materials Table S1).

## 4. Discussion

TTC is an acute heart failure condition that may resemble ACS or myocarditis due to its similar clinical symptoms, ECG alterations, and changes in standard laboratory parameters [22]. Currently, according to the Mayo criteria, coronary angiography is required to accurately differentiate between TTC and ACS [6]. Therefore, we aimed to investigate the hemogram parameters for differences among patients with TTC, ACS, or healthy controls. Thus far, the hemogram parameters and their ratios have shown prognostic value in TTC and ACS; however, studies depicting differences in the hemogram parameters among these two disease entities are lacking. Therefore, we sought to analyze these, not only for their differential diagnostic value but also to gain more information about the partly unknown pathophysiological background of TTC.

A significant increase in basophil levels and BLR in TTC patients compared to ACS and controls, with either a significance of *p* < 0.0001, was an interesting finding of our study and may indicate differential diagnostic value for distinction between TTC and ACS. Elevated levels of basophils in TTC patients, when compared to ACS and Ctrl, are an interesting finding, as mechanisms of autoimmunity following catecholamine excess have already been discussed to be involved in the pathophysiological process of TTC. A break in immunological tolerance to self-antigens may cause antigen-dependent autoimmunity response, and the persistent presence of the triggering self-peptide can sustain or even exacerbate autoreactivity. The heart is particularly susceptible to such a break in immunological self-tolerance because immunologic tolerance to myosin is missing. Even in healthy individuals, the immune system is easily activated if cardiac myosin is encountered under inflammatory conditions, such as being caused by catecholamine excess [23]. This theory is in accordance with our results and may confirm the role of basophils in the pathophysiology of TTC, as hypereosinophilic myofibers were observed in biopsies of TTC patients in preceding studies with peaks at day 6 [24]. As basophils are one of the main mediators of eosinophils, elevated basophil levels in TTC at baseline might perfectly suit previous study results. The role of basophils in the pathophysiology of TTC is underlined as there are several reports in which TTC followed anaphylactoid reactions [25]. Additionally, IgE-mediated cardiotoxic effects with inconspicuous coronaries in angiography have already been described in type I Kounis syndrome. Several parallels between Kounis syndrome and TTC have already been hypothesized [26]. However, differential blood testing to differentiate TTC from ACS patients has not been described in the literature. Basophils are secretors of serotonin, too. Our findings of statistically significantly higher basophil levels in TTC might be underlined, as higher serotonin levels in TTC patients have already been reported in a previous study. Laborde et al. even speculated that serotonin via its inotropic and apoptotic effect might be causal in the initiation of TTC [27]. The thesis of the relevance of serotonin and basophils in the pathophysiology of TTC may even be confirmed, as there are reports of the initiation of TTC after the onset of SSRIs [28]. The measurement of differential blood analysis might be preferred to the analysis of serotonin because results are faster accessible, easier analyzed, and less influenced by medications and renal function [29].

Catecholamines are known to decrease lymphocyte levels. As endogenous catecholamines are suspected to be the main triggers for TTC, low lymphocyte levels in TTC patients are in accordance with our expectations. Therefore, all three investigated ratios were significantly elevated in patients with TTC or ACS when compared with controls.

In contrast to basophils, monocyte levels were significantly higher in ACS patients compared to TTC patients. Catecholamines can induce regional myocardial edema, as often reported in TTC, and therefore may decrease monocyte levels by sequestration into the myocardium, although there is an increased release from the bone marrow into circulation [30]. Therefore, significantly lower monocyte counts in TTC compared to ACS may be explained.

Neutrophil counts and NLR were the highest in ACS patients, followed by a significant difference among healthy individuals. This association remained even statistically significant in multivariate analysis with correction for possible confounders, including serum creatinine, CRP, EF, age, and smoking. There was no significant difference between ACS and TTC. The results are in accordance with our expectations as neutrophils have been analyzed to be found in damaged myocardial structure and in atherosclerotic plaques.

Therefore, BLR may be useful in the distinction of TTC from ACS, whereas NLR differentiates ACS from Ctrl. Further large-scale studies are necessary to confirm the study results.

## 5. Conclusions

Basophil count and BLR are significantly increased in TTC patients when compared to ACS patients and healthy individuals. This interesting finding may confirm speculations about the partly unexplained pathophysiology of TTC. When considering clinical aspects, BLR may be useful in the distinction of TTC from ACS, whereas NLR may be helpful in the differentiation from ACS to Ctrl.

## 6. Limitations

Major limitations of the present study are the relatively small study cohort and the fact that patients were recruited in only one study center.

## Figures and Tables

**Table 1:** Baseline characteristics of patients in the three investigated groups. Abbreviations: BMI = body mass index, EF = ejection fraction, LDL = low density lipoprotein, CRP = C-reactive protein.

	TTC (*n* = 40)		ACS (*n* = 63)		Control (*n* = 68)		*p*-Value
	Median	IQR	Median	IQR	Median	IQR	
**Age (years)**	72.0	62.3–79.8	64.0	56.0–72.0	65.0	54.0–71.8	0.002
**BMI (kg/m^2^)**	26.7	22.1–29.3	27.8	25.9–31.3	27.7	23.8–30.7	0.034
**EF (%)**	40.0	35.0–45.8	50.0	45.0–66.8	67.0	62.8–74.0	<0.0001
**Creatinine (µmol/L)**	69.1	60.3–81.0	77.0	65.3–94.5	70.0	64.0–85.8	0.036
**LDL cholesterol (mg/dL)**	88.0	72.0–120.0	123.5	88.1–151.8	125.2	105.2–159.8	<0.0001
**CRP (mg/L)**	0.5	0.1–0.5	3.5	0.0–9.2	0.4	0.3–0.6	0.019
**Smoking**	11/40 (27.5%)		18/63 (28.6%)		28/68 (41.2%)		0.033
**Hypertension**	31/40 (77.5%)		53/63 (84.1%)		59/68 (86.8%)		0.450
**Diabetes**	4/40 (10.0%)		12/63 (19.0%)		19/68 (27.9%)		0.031
**Sex (female)**	36/40 (90.0%)		41/63 (65.1%)		12/68 (17.6%)		<0.0001

**Table 2:** White blood cell differentials and investigated ratios between the three groups.

	TTC (*n* = 40)		ACS (*n* = 63)		Control (*n* = 68)		*p*-Value
	Median	IQR	Median	IQR	Median	IQR	
**Leucocyte count (G/L)**	9.305	7.378–12.248	8.900	7.400–11.000	6.900	6.000–7.900	<0.0001
**Lymphocyte count (G/L)**	1.500	1.025–2.155	1.600	1.300–2.400	1.920	1.563–2.395	0.015
**Neutrophil count (G/L)**	5.230	3.840–10.480	6.400	4.680–8.300	4.000	3.300–4.700	0.002
**Monocyte count (G/L)**	0.610	0.485–0.720	0.800	0.590–0.850	0.500	0.400–0.693	<0.0001
**Basophil count (G/L)**	0.030	0.010–0.050	0.000	0.000–0.013	0.000	0.000–0.030	<0.0001
**Eosinophil count (G/L)**	0.070	0.010–0.125	0.055	0.000–0.200	0.140	0.100–0.208	0.140
**Neutrophil/Lymphocyte ratio**	3.204	2.125–6.266	3.822	2.296–5.929	2.095	1.706–2.667	<0.0001
**Basophil/Lymphocyte ratio**	0.019	0.011–0.029	0.000	0.000–0.008	0.000	0.000–0.014	<0.0001
**Monocyte/Lymphocyte ratio**	0.369	0.274–0.548	0.444	0.286–0.595	0.280-	0.198–0.361	<0.0001

**Table 3:** Bivariate correlation of baseline characteristics and investigated ratios. Abbreviations: NLR = Neutrophil/Lymphocyte ratio, BLR = Basophil/Lymphocyte ratio, MLR = Monocyte/Lymphocyte ratio, BMI = body mass index, EF = ejection fraction, CRP = C-reactive protein.

		BMI (kg/m^2^)	EF (%)	Age (Years)	CRP (mg/dL)	Creatinine (µmol/L)
**NLR**	*rs*	−0.002	−0.332	0.133	0.006	0.036
	*p-value*	0.980	0.001	0.158	0.947	0.676
**BLR**	*rs*	−0.143	−0.142	−0.123	−0.042	−0.127
	*p-value*	0.067	0.119	0.168	0.594	0.115
**MLR**	*rs*	0.109	−0.208	0.192	0.175	0.223
	*p-value*	0.164	0.021	0.030	0.024	0.005

## Data Availability

Data is available from the corresponding author upon reasonable request.

## Supplementary Materials

The following supporting information can be downloaded at: https://www.mdpi.com/article/10.3390/life12060788/s1.
