# Supplementary material for: Differences of Hemogram Parameters and Their Ratios among Patients with Takotsubo Syndrome, Acute Coronary Syndrome and Healthy Individuals
Source: Life (Basel). 2022 May 26;12(6):788. doi: 10.3390/life12060788 (PMC9224919; doi:10.3390/life12060788)
Supplement: Supplementary file 1 [file life-12-00788-s001.zip › life-1732832-supplementary.pdf]

Supplementary Table S1

	Univariate logistic regression dependent variable= ACS				M
	B (SE)	OR (95%CI)	R^2	p-value	B (SE)
Age	0.009 (0.017)	1.009 (0.98-1.04)	0.01	0.599	
Creatinine	0.018 (0.009)	1.018 (1.01-1.04)	0.04	0.036	
CRP	0.652 (0.218)	1.920 (1.25-2.94)	0.29	0.003	
BMI	0.064 (0.037)	1.066 (0.99-1.15)	0.03	0.080	
EF	-0.089 (0.022)	0.915 (0.88-0.95)	0.24	<0.0001	
Hypertension	-0.213 (0.497)	0.808 (0.31-2.14)	0.00	0.669	
Diabetes	0.724 (0.421)	2.062 (0.90-4.70)	0.02	0.085	
Smoking	0.859 (0.385)	2.360 (1.11-5.02)	0.04	0.026	
NLR	0.728 (0.166)	2.072 (1.50-2.87)	0.24	<0.0001	
BLR	-0.600 (3.896)	0.549 (0.00-1135.96)	0.00	0.878	
MLR	0.601 (0.454)	1.823 (0.75-4.44)	0.02	0.186	

	Univariate logistic regression dependent variable= TTC				M
	B (SE)	OR (95%CI)	R^2	p-value	B (SE)
Age	0.077 (0.023)	1.080 (1.03-1.13)	0.12	0.001	
Creatinine	-0.001 (0.010)	0.999 (0.98-1.02)	0.00	0.935	
CRP	0.636 (0.347)	1.888 (0.96-3.73)	0.08	0.067	
BMI	-0.059 (0.045)	0.942 (0.86-1.03)	0.02	0.183	
EF	-0.315 (0.076)	0.730 (0.63-0.85)	0.64	<0.0001	
Hypertension	-0644 (0.521)	0.525 (0.19-1.46)	0.01	0.217	
Diabetes	-0.657 (0.616)	0.519 (0.16-1.73)	0.01	0.286	
Smoking	-0.076 (0.451)	0.927 (0.38-2.25)	0.00	0.867	
NLR	0.408 (0.143)	1.504 (1.14-1.99)	0.18	0.004	
BLR	6.675 (6.208)	791.986 (0.00-15236.78)	0.02	0.282	
MLR	0.287 (0.398)	1.333 (0.61-2.91)	0.01	0.470	

Multivariate logistic regression
dependent variable= ACS

OR (95%CI)	R^2	p-value

Multivariate logistic regression
dependent variable= TTC

OR (95%CI)	R^2	p-value
